# Digital interventions for socially isolated youth: exploring clinical and cognitive outcomes from an Italian multicentric study

**DOI:** 10.1192/j.eurpsy.2026.10326

**Published:** 2026-07-31

**Authors:** M. G. Rossetti, M. Bellani, The SOLITAIRE group

**Affiliations:** 1 Department of Neurosciences, Biomedicine and Movement Sciences, University of Verona; 2 UOC Psichiatria B, Azienda Ospedaliera Universitaria Integrata, Verona, Italy

## Abstract

**Background:**

Social Isolation (SI) in youth is an increasingly prevalent condition that can evolve into severe forms of withdrawal, such as Hikikomori syndrome. SI often acts as a precursor or a core symptom of various psychiatric conditions, including depression, social anxiety, and psychosis. Traditional in-person interventions often struggle to engage these individuals due to the inherent social barriers associated with the condition. Consequently, there is an urgent need for flexible, digital-based therapeutic approaches.

**Objective:**

The SOLITAIRE project aims to evaluate the feasibility and preliminary efficacy of a multimodal digital intervention for adolescents and young adults experiencing moderate to severe SI. The study specifically investigates the impact of remote Cognitive Behavioural Therapy (CBT), both alone and in combination with online Cognitive Remediation (CR), on social isolation, associated clinical symptoms, and neurocognitive functioning.

**Methods:**

This multicentric clinical trial involves adolescents and young adults (aged 11-45) recruited across two Italian clinical sites. Inclusion is based on a moderate-to-severe SI profile, assessed via the 25-item Hikikomori Questionnaire (HQ-25). Participants are randomised to receive either tele-CBT alone or a synergistic CBT+CR intervention over approximately eight weeks. Clinical outcomes (e.g., SI, depression, social anxiety, alexithymia, and impulsivity) and cognitive performance (attention, memory, and social cognition) are assessed at baseline (T0) and post-treatment (T1).

**Results:**

Recruitment for the SOLITAIRE project began in June 2023 and is scheduled for completion in May 2026. To date, 51 participants completed the study, and further participants are currently undergoing assessment and intervention. Preliminary baseline data (T0) indicate that participants exhibit high levels of social isolation (HQ-25), often accompanied by varying degrees of depressive symptoms, alexithymia, and social anxiety. Longitudinal analyses to compare pre- and post-treatment outcomes (T0 vs. T1) and to evaluate the specific added value of CR are currently underway. Updated statistical findings, including the latest available data on treatment efficacy and dropout rates, will be presented at the conference.

**Conclusion:**

While the project is still ongoing, the SOLITAIRE study represents a significant effort to address the unmet needs of socially isolated youth through accessible digital tools. Based on the preliminary results of this study, it will be possible to implement larger randomised controlled trials to further validate and refine this approach, ultimately contributing to the development of more effective, personalised remote interventions to promote real-world social reintegration and improve the quality of life for this vulnerable population.

**Disclosure of Interest:**

None Declared

